# Raman spectroscopy‐based diagnostics of water deficit and salinity stresses in two accessions of peanut

**DOI:** 10.1002/pld3.342

**Published:** 2021-08-20

**Authors:** Rohini Morey, Charles Farber, Bill McCutchen, Mark D. Burow, Charles Simpson, Dmitry Kurouski, John Cason

**Affiliations:** ^1^ Department of Biochemistry and Biophysics Texas A&M University College Station Texas USA; ^2^ Texas A&M AgriLife Research Stephenville Texas USA; ^3^ Texas A&M AgriLife Research Lubbock Texas USA; ^4^ Department of Biomedical Engineering Texas A&M University College Station Texas USA

**Keywords:** groundnuts, peanuts, plants, Raman spectroscopy, salinity stress, water deficit stress

## Abstract

Water deficit and salinity are two major abiotic stresses that have tremendous effect on crop yield worldwide. Timely identification of these stresses can help limit associated yield loss. Confirmatory detection and identification of water deficit stress can also enable proper irrigation management. Traditionally, unmanned aerial vehicle (UAV)‐based imaging and satellite‐based imaging, together with visual field observation, are used for diagnostics of such stresses. However, these approaches can only detect salinity and water deficit stress at the symptomatic stage. Raman spectroscopy (RS) is a noninvasive and nondestructive technique that can identify and detect plant biotic and abiotic stress. In this study, we investigated accuracy of Raman‐based diagnostics of water deficit and salinity stresses on two greenhouse‐grown peanut accessions: tolerant and susceptible to water deficit. Plants were grown for 76 days prior to application of the water deficit and salinity stresses. Water deficit treatments received no irrigation for 5 days, and salinity treatments received 1.0 L of 240‐mM salt water per day for the duration of 5‐day sampling. Every day after the stress was imposed, plant leaves were collected and immediately analyzed by a hand‐held Raman spectrometer. RS and chemometrics could identify control and stressed (either water deficit or salinity) susceptible plants with 95% and 80% accuracy just 1 day after treatment. Water deficit and salinity stressed plants could be differentiated from each other with 87% and 86% accuracy, respectively. In the tolerant accessions at the same timepoint, the identification accuracies were 66%, 65%, 67%, and 69% for control, combined stresses, water deficit, and salinity stresses, respectively. The high selectivity and specificity for presymptomatic identification of abiotic stresses in the susceptible line provide evidence for the potential of Raman‐based surveillance in commercial‐scale agriculture and digital farming.

## Highlights

We show that Raman spectroscopy can be used for presymptomatic diagnosis of water deficit and salinity stresses in two peanut accessions. We found that the accuracy of stress detection and identification directly depends on the plant sensitivity to the stress imposed.

## 1 INTRODUCTION

Peanut (*Arachis hypogaea* L.) is an allotetraploid (2*n* = 4*x* = 40) that has been cultivated for thousands of years (Singh & Simpson, 1994). Today, it is grown throughout the temperate and tropical parts of the world and is an important international crop (Kochert et al., 1991; Krapovickas & Gregory, 1994). Areas of production range from subsistence farming to large‐scale commercial operations on all continents except Antarctica (ICRISAT, 2018). Total worldwide peanut production was estimated to be 49 million metric tons (MT) in 2021 (USDA, 2021b). Leading countries in production are China (18.2 million MT), India (6 million MT), and Nigeria (4.4 million MT) (USDA, 2021b).

Peanuts, widely known as groundnuts are used in many ways: over 50% of worldwide production is crushed for use as oil (TPF, 2018). Other uses include peanut cake and meal and direct consumption or as an ingredient in foods (TPF, 2018). Use varies by country: most peanuts in the United States are used in peanut butter, confectionary products, or are exported (NPB, 2018).

In the United States, approximately 653,900 ha of peanuts were harvested in 2020, with an average yield of 4.5 metric tons/ha (USDA, 2021a). The estimated farm value of US production is more than one billion US dollars, with peanut being listed as the 12th most valuable cash crop in the United States (TPF, 2018). Peanut production is concentrated in the Southern United States, from the eastern seaboard to New Mexico. Georgia is the leading peanut producing state followed by Florida, Alabama, and Texas (USDA, 2016).

Crops experience a variety biotic and abiotic stresses that can decrease growth and productivity (Farber, Mahnke, et al., 2019). Although plant diseases can cause up to 30% loss of the crop yield worldwide, losses associated with abiotic stresses, such as water deficit, nutrient deficiencies and salinity, may reach 70% (Pandey et al., 2017).

Drought is a growing problem not only in arid and semi‐arid climate zones but also in areas with continental climate (Waraich et al., 2012). In 2012, during one of the worst droughts in recent US history, the USDA reported that approximately $14.5 billion in federal insurance was paid to growers for drought‐associated losses, 83% of all insurance paid out that year (Nelson, 2017). The increased frequency of drought events is cause for concern because it has been estimated that up to 80% of the peanut production in the world is centered in areas that use no irrigation and are subject to unpredictable droughts (Wright & Nageswara Roa, 1994). The High Plains of Texas are an excellent example of the increasing concern over drought and groundwater levels. Irrigation water coming from the Ogallala aquifer is used throughout most of the region. Chaudhuri and Ale reported estimates that 90% of the water pumped out of the Ogallala aquifer in Texas is for the purpose of irrigation (Chaudhuri & Ale, 2014). The United States Geological Survey has estimated that groundwater use in the High Plains ranges from 10.7–19.9 million liters per year (TWDB, 2012). This represents an average irrigation rate of 213.6 to 411.5 mm/year (USGS, 2011). It has been estimated that median water levels of the Ogallala aquifer in the Texas Panhandle dropped from 25 to 67 m in the 70 years since irrigated agriculture has become common (Chaudhuri & Ale, 2014).

Soil salinity is a global problem, especially in numerous developing countries as well as Western Texas (Trostle, 2016). High osmotic pressure under salinity stress in the soil prevents water and mineral uptake by plants. This drastically reduces crop yields and, ultimately, the productivity in the high salinity areas. Research in Egypt showed an approximately 50% reduction in yield of peanuts (El‐RheemKh, & A. and Zaki, S.‐n.S., 2015). Salinity stress is extremely difficult to treat and is usually planned for by measuring soil salinity before planting (Bauder et al., 2004). When such testing is not possible, identifying salinity stress after planting could allow growers to plan out their long‐term response strategies.

Timely diagnostics of drought can be used for site‐ and dose‐specific administration of water to the field which allows for preservation of the crop yield (Food and Agriculture Organization of the United Nations, 2009). Imaging methods, including thermography, hyperspectral, and RGB, can be used to diagnose water deficit and salinity stresses by detecting changes in the color, texture, or temperature of the plant. If measured from a plane or unmanned aerial vehicle (UAV), these imaging methods can survey entire fields (Baena et al., 2017). However, none of imaging approaches achieved broad application in agriculture due to their poor specificity, complex data analysis, and long image processing times.

Raman spectroscopy (RS) probes the chemical structure of samples through inelastic light scattering (Farber, Mahnke, et al., 2019). In a Raman experiment, laser light is shined on the sample. The light interacts with the sample. Photons that change direction and energy (inelastically scattered photons) are collected with a spectrometer and are plotted based on their energy relative to the initial laser light. The change, or shift, in energy (also known as the Raman shift) is dependent on the identities of chemical groups in the sample, making Raman sensitive to chemical structure.

Our group showed that RS could be used for confirmatory diagnostics of fungal diseases on corn, wheat, and sorghum (Egging et al., 2018, Farber & Kurouski, 2018). We also demonstrated that RS was capable of detection of viral diseases of wheat and rose, as well as the presence of bacteria that cause Huanglongbing (HLB or citrus greening) on citrus trees (Farber, Shires, et al., 2019; Sanchez, Pant, Irey, et al., 2019; Sanchez, Pant, Xing, et al., 2019). This approach is based on detecting changes in the host tissue associated with the infection. Because these changes are pathogen‐specific, RS has species‐level sensitivity. Similar metabolic changes are observed upon abiotic stresses (Altangerel et al., 2017; Gupta et al., 2020; Sanchez, Ermolenkov, Biswas, et al., 2020). Detection and identification of these changes in rice enabled highly accurate identification of nitrogen (N), phosphorus (P), and potassium (K) deficiencies with 85% accuracy as early as 2 days after stress introduction (Sanchez, Ermolenkov, Biswas, et al., 2020).

The hand‐held nature of the spectrometer used in our previous studies suggests that spectroscopic analysis of plants can be performed directly in the field or a greenhouse (Farber, Sanchez, & Kurouski, 2020; Farber et al., 2021; Farber, Sanchez, Rizevsky, et al., 2020; Farber, Shires, et al., 2019; Sanchez, Ermolenkov, Biswas, et al., 2020; Sanchez, Ermolenkov, Tang, et al., 2020). Expanding upon these findings, we investigated the accuracy of the Raman‐based approach in the detection of water deficit and salinity stresses in two peanut accessions: Tamrun OL11, a water deficit stress‐susceptible line, and TxL100225‐05‐07, a tolerant line developed in Lubbock, TX (Baring et al., 2013). Both accessions were grown in the greenhouse, subjected to water deficit and salinity stresses, and analyzed using RS at daily for 5 days after introduction of the stress.

## 2 EXPERIMENTAL PROCEDURES

### 2.1 Plant materials and set up

A replicated, imposed water deficit and salinity study was conducted during the spring of 2020 at the greenhouses of the Texas A&M AgriLife Research and Extension Center at Stephenville. The study was conducted in an Ickes‐Braun Glasshouses (IBG) greenhouse operating on a Wadsworth Step‐50 temperature control system. The system operated where the heaters activate if the temperature drops below 21°C, and the cooling system activates if the temperature exceeds 32°C.

The study contained one drought tolerant breeding line, TxL100225‐05‐07 (TX225), and drought‐susceptible line, Tamrun OL11 (OL11), from the Texas A&M AgriLife Research Peanut Breeding program. The lines were chosen based on 2018 field data from Dr. Mark Burow in which breeding lines were tested for yield under water‐reduced irrigation at 25% ET replacement. Analysis revealed that TX225 performed in the top statistical grouping with an average yield of 2,826 kg/ha, while OL11 was the lowest performing runner cultivar currently in commercial production with an average yield of 1425.8 kg/ha. In this present study, 25 plants were grown of each genotype for three distinct physiological states, which were well watered, water deficit stressed, and a salinity stressed state (240‐mM NaCl). Seeds were pregerminated in a Stults germinator beginning on April 4, 2020 and planted on April 7, 2020. The germinator operated on a 14‐h photoperiod at a light temperature of 32°C and a dark temperature of 24°C. Plants were planted in 24‐cm diameter (8‐L volume) plastic pots with Windthorst fine sandy loam soil where they were watered daily as needed until sampling. Plants were sampled with a handheld Resolve Agilent Raman Spectrometer at the greenhouse complex head‐house. Collection of leaf tissue began at 76 days after planting (DAP) at which time water deficit and salinity stress were imposed for 5 days. Although the hand‐held Raman spectrometer used in our study allows for fully noninvasive and nondestructive analysis of plants, in the reported experiments, leaves were detached from the plants, wrapped to limit desiccation, and transported to the portable instrument for immediate spectroscopic analysis. This was done to minimize exposure of the personnel to extreme heat conditions in the greenhouse and to ease instrument fixation at the target. Imposed water deficit and salinity stress was started on Day 76 and continued until Day 80. Water deficit treatments received no irrigation for the duration of the sampling and salinity treatments received 1.0 L of 240‐mM salt water per day for the duration of the sampling. Main axis leaflets were sampled on five consecutive days between 8:00 a.m. and 12:00 a.m. Tissue samples were taken from the same fully expanded tetrafoliate leaves of the mainstem of each of the biological replicates for both genotypes in each of all three physiological states. To minimize the biochemical differences associated with location on the plant, on each day of sampling, a different leaf on the main axis was selected for sampling starting with the third fully expanded leaf on D1, fourth on D2, fifth on D3, sixth on D4, and seventh on D5. To minimize differences in diurnal plant cycles, collection of all tissue occurred at the same time each day with each day's sampling taking approximately 4 h to complete.

### 2.2 Raman spectroscopy

Raman spectra were collected with a hand‐held Agilent Resolve spectrometer equipped with an 830‐nm laser source. The following experimental parameters were used for all collected spectra: 1‐s acquisition time, 495‐mW power, and baseline spectral subtraction by device software. Previously reported experimental results demonstrated absence of photodegradation of plant material at these experimental conditions (Sanchez, Pant, Irey, et al., 2019). Fifty spectra were collected from each group of plants at a given time point. Spectra shown in the manuscript are baseline corrected by the instrument software without smoothing.

### 2.3 Multivariate data analysis

PLS Toolbox (Eigenvector Research Inc.) was used for statistical analyses of the collected Raman spectra. First, multiplicative signal correction based on the mean was applied to all data. Next, the second derivative was taken of the Raman spectra with a filter width of 51 and polynomial order 3. Finally, the spectra were smoothed with a 15‐point window then area normalized. Partial least squares discriminant analysis (PLS‐DA) was performed to differentiate between the experimental classes and identify spectral regions that best explained separation between the classes. Our own experimental results, as well as findings reported by other groups, show that PLS‐DA performs equally well or better than other chemometric methods, such as linear discriminant analysis (LDA) or soft independent modeling by class analogy (SIMCA) (Farber et al., 2021; Lee et al., 2018; Sanchez, Pant, Irey, et al., 2019; Sanchez, Pant, Mandadi, & Kurouski, 2020; Shashilov & Lednev, 2010). Therefore, we have selected PLS‐DA for statistical analysis of spectra collected in this study. All Raman shifts in the original spectra (350–2,000 cm^−1^) were used to build the reported models.

### 2.4 Hierarchical models

A two‐tier hierarchical modeling approach was employed to differentiate the three treatments. The first model (Hierarchical Model 1) differentiated the healthy (control, C) spectra from the sick (water deficit or salinity stressed, S) spectra. The second model (Hierarchical Model 2) then differentiated the water deficit (DR) spectra from the salinity stressed (Sal). The models were cross‐validated using the original dataset with the venetian blinds method using 10 data splits and one sample per blind. The results of model cross validation are reported in Tables 2 and 3. The accuracy reported in this study is the true positive rate, which is (for a given group) the number of spectra correctly assigned to the group divided by the total of spectra in that group.

## 3 RESULTS

**FIGURE 1:**
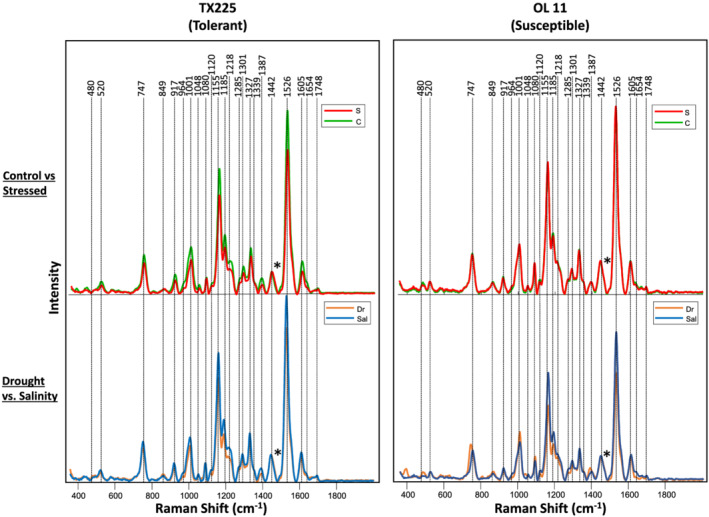
Raman spectra collected from leaves of control (“C”, green) and stressed (average of both drought and salinity stress, “S”, red) peanut lines (top panels). Spectroscopic signatures of water deficit stressed (“Dr”, orange) and salinity stressed (“Sal”, blue) plants (bottom panel). Spectra normalized on CH_2_ vibrations (1,442 cm^−1^) present in nearly all classes in biological molecules (marked by asterisks [*])

Spectra collected from leaves of peanuts exhibited vibrational bands assigned to pectin (747 cm^−1^), cellulose (480, 520, 917, 1,048, and 1,080 cm^−1^), xylan (1,185 cm^−1^), carotenoids (1,000, 1,155, and 1,525 cm^−1^), phenylpropanoids (1,605 cm^−1^), protein (1,654 cm^−1^), and aliphatic vibrations (1,218, 1,285, 1,327, 1,339, 1,387, and 1,442 cm^−1^) (Figure 1 and Table 1). These assignments were made based on the previous studies reported by our and other research groups (Altangerel et al., 2017; Farber et al., 2021; Farber & Kurouski, 2018; Farber, Sanchez, & Kurouski, 2020; Farber, Sanchez, Rizevsky, et al., 2020; Gupta et al., 2020; Mandrile et al., 2019; Payne & Kurouski, 2021; Sanchez, Ermolenkov, Tang, et al., 2020; Sanchez, Pant, Xing, et al., 2019). We found that spectra collected from stressed (symptomatic, D5) TX225 plants exhibited lower intensities of vibrational bands that originated from pectin, cellulose, xylan, aliphatic vibrations, and carotenoids compared to those of control plants (Figure 1). In contrast, only small spectral changes were observed in the susceptible line. We found that water deficit and salinity stresses have different spectroscopic fingerprints in both TX225 and OL11. In TX225, water deficit stress can be characterized by the small increase in the intensities of 849 and 1,442 cm^−1^, which could be assigned to pectin and aliphatic vibrations. The susceptible OL11 variety exhibited much stronger spectroscopic changes associated with water deficit, including strong decreases in carotenoid (1,155, 1,185, and 1,526 cm^−1^), phenylpropanoid (1,605 cm^−1^), and aliphatic vibration intensities (1,301, 1,387 and 1,442 cm^−1^).

**TABLE 1:** Vibrational bands and their assignments for spectra collected from control peanuts, as well as plants exposed to water deficit and salinity stresses

Band (cm^−1^)	Vibrational mode	Assignment
480	C‐C‐O and C‐C‐C deformations; related to glycosidic ring skeletal deformations δ(C‐C‐C)+τ(C‐O) scissoring of C‐C‐C and out‐of‐plane bending of C‐O	Carbohydrates (Almeida et al., 2010)
520	ν(C‐O‐C) Glycosidic	Carbohydrates (Edwards et al., 1997, Pan et al., 2017)
747	γ(C–O‐H) of COOH	Pectin (Synytsya et al., 2003)
849–853	(C_6_–C_5_–O_5_–C_1_–O_1_)	Pectin (Engelsen & Nørgaard, 1996)
917	ν(C‐O‐C) in‐plane, symmetric	Cellulose and phenylpropanoids (Edwards et al., 1997)
964–969	δ (CH_2_)	Aliphatics (Cabrales et al., 2014, Yu et al., 2007)
1,000–1,005	In‐plane CH_3_ rocking of polyene Aromatic ring of phenylalanine	Carotenoids (Schulz et al., 2005) and protein
1,048	ν(C‐O) + ν(C‐C) + δ(C‐O‐H)	Cellulose and phenylpropanoids (Edwards et al., 1997)
1,080	ν(C‐O) + ν(C‐C) + δ(C‐O‐H)	Carbohydrates (Almeida et al., 2010)
1,115–1,119	Sym ν(C‐O‐C), C‐O‐H bending	Cellulose (Edwards et al., 1997)
1,155	C‐C stretching; v(C‐O‐C), v(C‐C) in glycosidic linkages, asymmetric ring breathing	Carotenoids (Schulz et al., 2005) and carbohydrates (Wiercigroch et al., 2017)
1,185	ν(C‐O‐H) next to aromatic ring+σ (CH)	Carotenoids (Schulz et al., 2005)
1,218	δ(C‐C‐H)	Carotenoids (Schulz et al., 2005) and xylan (Agarwal, 2014)
1,265	Guaiacyl ring breathing, C‐O stretching (aromatic); ‐C=C‐	Phenylpropanoids (Cao et al., 2006) and unsaturated fatty acids (Jamieson et al., 2018)
1,286	δ(C‐C‐H)	Aliphatics (Yu et al., 2007)
1,301	δ(C‐C‐H) + δ(O‐C‐H) + δ(C‐O‐H)	Carbohydrates (Almeida et al., 2010, Cael et al., 1975)
1,327	δCH_2_ bending	Aliphatics, cellulose, and phenylpropanoids (Edwards et al., 1997)
1,339	ν(C‐O); δ(C‐O‐H)	Carbohydrates (Almeida et al., 2010)
1,387	δCH_2_ bending	Aliphatics (Yu et al., 2007)
1,442	δ (CH_2_)	Aliphatics (Yu et al., 2007)
1,515–1,535	‐C=C‐ (in‐plane)	Carotenoids (Adar, 2017, Devitt et al., 2018, Rys et al., 2014)
1,606–1,632	ν(C‐C) aromatic ring+σ (CH)	Phenylpropanoids (Agarwal, 2006, Kang et al., 2016)
1,654–1,660	‐C=C‐, C=O stretching, amide I	Unsaturated fatty acids (Jamieson et al., 2018) and proteins (Devitt et al., 2018)
1,682	COOH	Carboxylic acids (Sanchez, Filter, Baltensperger, & Kurouski, 2020)
1,748	C=O stretching	Esters, aldehydes, carboxylic acids, and ketones (Colthup et al., 1990)

Next, we constructed PLS‐DA models to differentiate control, water deficit, and salinity stresses on OL11 and TX225 based on the spectra of their leaves. We built two models for each plant accession at each time point. The first determined whether the plant was stressed (control vs. combined stressed), while the second determined which stress it was (water deficit vs. salinity). Our results demonstrated that in OL11 at the first time point, the control and combined stress groups can be differentiated from each other with 95.6% and 80.8% accuracy, respectively, while water deficit and salinity can be differentiated with 87.5% and 86.3% accuracy, respectively (Table 2 and Figure 1). No visual signs of stress such as loss of turgor, leaf curling, or leaf yellowing were observed at this timepoint. We also found that as the experiment progressed, the accuracy of classification typically increased. Specifically, at D4, water deficit and salinity stress can be distinguished from each other with 89.1% and 91.8% accuracy, whereas at D5, the accuracy reaches 94.0% and 91.5%. In both tolerant and susceptible plants, visual signs of stress appeared on Days 2 and 3 for drought and salinity, respectively. This may be because stress occurs more quickly under greenhouse conditions than in the field.

**TABLE 2:** PLS‐DA based accuracy of prediction of the two model types for the OL11 (susceptible) peanut line

	Hierarchical model 1		Hierarchical model 2	
Day	Control	Stressed	Water deficit	Salinity
1	95.6	80.8	87.5	86.3
2	91.5	85.8	72.9	79.2
3	97.9	86.8	86.0	89.8
4	91.3	81.0	89.1	91.8
5	80.0	72.8	94.0	91.5

Abbreviation: PLS‐DA, partial least squares discriminant analysis.

**FIGURE 2:**
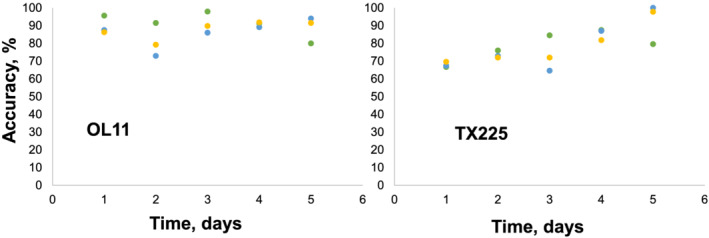
Accuracy of prediction of control (green), water deficit (blue) and salinity (yellow) stresses on susceptible (OL11) and tolerant (TX225) peanut lines

Prior to visual signs of stress, the accuracy classification by stress status was substantially lower for TX225. At D1, the accuracy of differentiating the control and combined stress groups was 66.7% and 65.6%, respectively. Water deficit and salinity identification rates were 67.4% and 69.6%, respectively (Table 3 and Figure 2). As observed with OL11, the prediction accuracy generally improved over the course of the experiment. However, at D4, control/combined stress differentiation showed a great decrease in performance with accuracies of 87.5% and 58.9%, respectively. The water deficit/salinity continued to perform well, in contrast, showing 87% and 81.8% accuracy, respectively. At D5, accuracy of water deficit stress identification for TX225 was 100%, whereas salinity stress could be correctly predicted with 97.8% accuracy.

**TABLE 3:** PLS‐DA based accuracy of prediction of the two model types for the TX225 (tolerant) peanut line

	Hierarchical model 1		Hierarchical model 2	
Day	Control	Stressed	Water deficit	Salinity
1	66.7	65.6	67.4	69.6
2	76.0	73.9	72.9	72.0
3	84.5	89.3	64.6	72.0
4	87.5	58.9	87.0	81.8
5	79.6	80.2	100	97.8

Abbreviation: PLS‐DA, partial least squares discriminant analysis.

**FIGURE 3:**
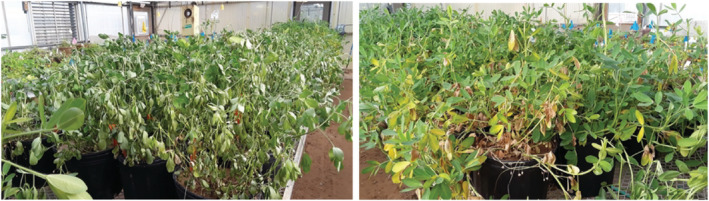
A picture showing plants exhibiting severe water deficit (left) and salinity stress (right) including leaf curing, loss of turgor, and leaf yellowing on Day 5 of sampling

Our results demonstrate that in the late stages (symptomatic plants), water deficit, and salinity stresses can be correctly identified with ~90% accuracy in both tolerant and susceptible plants. However, in early stages, the model performed poorly with the tolerant dataset. This complicates the differentiation of stressed and unstressed plants in these lines. Because these varieties demonstrate such drastic differences in response, development of a universal chemometric model for identification of stress without regard to genetic background is not possible. When the data from each line were combined to make a single model to classify by stress, the accuracy was low. Although this high sensitivity of RS substantially complicates identification of stresses, it can be used to identify accessions. This identification is based on detection of differences in biochemical profiles of different peanut varieties (Farber, Sanchez, Rizevsky, et al., 2020). We found that on average, OL11 and TX225 could be identified with 81.4% accuracy. We previously demonstrated that such Raman‐based phenotyping can be used not only for identification of different peanut accessions but also for digital breeding and selection of plants (Farber, Sanchez, Rizevsky, et al., 2020). We also demonstrated that in potatoes, the performance of models for identifying biotic stress (zebra chip disease, ZC) in tubers was dependent on the variety: a model calibrated with one variety of potatoes could not accurately identify ZC‐associated changes in potatoes of another variety (Farber et al., 2021).

## 4 DISCUSSION

This study demonstrated the power of RS for label‐free, noninvasive, and nondestructive detection and identification of water deficit and salinity stresses in peanuts. Stress in greenhouses occurs more quickly than under field conditions. Our results showed that in early stages (D1), these stresses could be predicted with high accuracy only on susceptible plants, whereas in the later stages (D5), Figure 3, accurate identification of water deficit and salinity can be achieved on both susceptible and tolerant peanut accessions. These results are not totally unexpected since the water deficit tolerant germplasm used in this study was specifically developed to withstand water deficit stress better than the commercial check which was specifically bred to be produced under full irrigation. These results suggest that the tolerant breeding line was able to maintain normal plant functions for a longer time as the water deficit and salinity stress were applied. As the two accessions were subjected to the stressors for longer periods of time, RS was able to detect the changes occurring in both the tolerant and susceptible germplasm. In previous studies, it has been suggested that increasing the sample size can improve model accuracy. Although drought tolerant cultivars are preferred, RS has the potential to detect early plant stress on a specific cultivar by incorporating enough calibration scans into the model. For example, when our group attempted to classify peanut leaves by genotype, we found moderate success when acquiring 70 spectra per genotype (Farber, Sanchez, Rizevsky, et al., 2020). Different analysis methods could also improve the result. Liu and colleagues found that convolutional neural networks, which are more computational intensive than PLS‐DA, could classify spectra of over 1,600 different minerals with a few as 1–40 spectra per group (Liu et al., 2017).

The ability to detect the initial signs of stress could play a very important role is the management of a crop in the field. Once plants exhibit visual signs of water deficit or salinity stress, the potential yield has already decreased. Early detection of these stresses is therefore desirable. Precise management of irrigation on a commercial scale can also lead to increased yields and savings on irrigation costs. Incorporation of RS into a UAV platform with real‐time or rapid turnaround of results could potentially be part of an integrated, automated irrigation management system.

The observed decrease in carotenoid‐associated band intensity in water deficit TX225 leaves may be physiologically relevant (Havaux, 2013). Biotic and abiotic stresses activate enzymatic oxidation of neoxanthin that yields abscisic acid, a hormone that enhances plant resistance to such stresses (Nambara & Marion‐Poll, 2005). β‐Carotene oxidation and cleavage by reactive oxygen species (ROS) lead to formation of β‐lonone, β‐cyclocitrals that can protect the plant against insects (Havaux, 2013; Nambara & Marion‐Poll, 2005). Thus, decreases in carotenoid concentration may be associated with increases in ROS concentration (Yu et al., 2007). The observed decrease in intensity of Raman bands associated with phenylpropanoids may be partially explained by a decrease in the concentration of *p*‐coumaryl and coniferyl alcohols, the precursors of H‐ and G‐lignins (Chishaki & Horiguchi, 1997). Alternatively, this may be due to a decreased concentration of kaempferol, quercetin, or isorhamnetin (Stewart et al., 2001). Spectroscopic analysis of these compounds reported by Jurasekova and co‐authors indicates that quercetin's phenolic vibrational band was at 1,610 cm^−1^, whereas kaempferol's phenolic vibrational band was at 1,604 cm^−1^ (Jurasekova et al., 2006). Based on this experimental evidence, the observed intensity decrease may be associated with a decrease in concentration of kaempferol in peanut leaves.

Having a tool to rapidly identify when plants are responding to stress could enable faster selection of drought and salinity tolerant cultivars. For example, RS could be used to rapidly survey F_2_ or F_3_ plants for those with the most favorable stress responses bring to field trials. Considering the high sensitivity of RS for the diagnostics of biotic and abiotic stresses in plants (Egging et al., 2018, Farber & Kurouski, 2018, Farber, Mahnke, et al., 2019, Farber et al., 2019b, Sanchez, Pant, Irey, et al., 2019, Sanchez, Pant, Xing, et al., 2019, Sanchez, Ermolenkov, Tang, et al., 2020), one can expect that as more scans are added to the libraries, this spectroscopic approach will have far‐reaching implications in various disciplines, from basic plant biology and pathology to agriculture and horticulture.

The Raman‐based diagnostics approach could be performed directly by farmers or implemented as a service. This would involve development of portable spectrometers and integrated spectral libraries. If the farmer chose to purchase the device and libraries themselves, they may need to calibrate the device for their crop varieties depending on whether the crop is included in the prebuilt library. Alternatively, as a service, a technician would come with a device and perform the calibration. Because Raman is sensitive to both stress and time, it may be possible that an initial calibration is required the season before the device can begin accurately classifying. This would be based on how Raman spectra of plants can change over their lifespans. In either case, once the instrument is initially calibrated, it should be ready for prediction in the following seasons.

With the calibrated instrument, whether handheld or mounted on a UAV, the farmer would be able to conduct the stress analysis themselves. The device would scan individual plants and send the results to a local laptop that would indicate the probability that different stresses were present on the plant. The system could alternatively be operated by a technician who would prepare a report indicating locations of stress throughout the field, their probabilities of having certain stresses, and suggested treatments. Either scenario would enable the farmer to respond to shifting environmental conditions in different parts of their field, applying differential treatments to areas in need. Although the description of Raman‐based stress diagnostics may look futuristic, our group is currently working on all technical aspects of this approach to make it a reality.

## CONFLICT OF INTEREST

The authors declare that they have no competing interests.

## Supporting information

**Data S1.** Supporting Information
