# Supplementary material for: Raman spectroscopy‐based diagnostics of water deficit and salinity stresses in two accessions of peanut
Source: Plant Direct. 2021 Aug 20;5(8):e342. doi: 10.1002/pld3.342 (PMC8377774; doi:10.1002/pld3.342)
Supplement: Supplementary file 1 — Data S1. Supporting Information [file PLD3-5-e342-s001.pdf]

Raman Spectroscopy-Based Diagnostics of Water Deficit and Salinity Stresses in Two Accessions of Peanut – Response to Editor Comments

L. 32: Please change to "RS and chemometrics could identify control and stressed plants of the susceptible accession (either water deficit or salinity) with 95 and 80% accuracy."

We have changed the sentence to read:

"RS and chemometrics could identify control and stressed (either water deficit or salinity) susceptible plants with 95 and 80% accuracy just one day after treatment."

L. 35: Since the lower percentage of accuracy for the tolerant cultivar was added here, the next sentence needs rephrasing; 65 to 69% is arguably not highly specific or sensitive. Please remove the word "great" from line 37.

We have changed the sentence to read:

The high selectivity and specificity for pre-symptomatic identification of abiotic stresses in the susceptible line provides evidence for the potential of Raman-based surveillance in commercial-scale agriculture and digital farming.

L. 170: Replace "(control, H)" with "(control, C)".

We have addressed this. Thank you.

L. 188: Replace "healthy" with "control"

We have addressed this. Thank you.

L. 216/217: Specify on which day the tolerant lines showed visible signs of stress.

We have added the following information to address this.

In both tolerant and susceptible plants, visual signs of stress appeared on days 2 and 3 for drought and salinity, respectively. The tolerant plants may have showed stress responses sooner because the stress occurred more quickly under greenhouse conditions than in field conditions.

L. 273 onwards: Provide a reference here, and how many scans would be needed according to those references.

We have added a passage around line 273 explaining how different sampling sizes or analysis methods could improve the prediction results.

L. 274: Rephrase to: "Although drought tolerant cultivars are preferred, RS has the potential to

detect early plant stress on a specific cultivar by incorporating a sufficient number of calibration scans."

We have rephrased as requested.

L. 90 & 292: Formatting error (dot before bracket, no space).

Thank you for pointing these out. They have been addressed.
